# Nucleation and Cascade Features of Earthquake Mainshock Statistically Explored from Foreshock Seismicity

**DOI:** 10.3390/e21040421

**Published:** 2019-04-19

**Authors:** Masashi Kamogawa, Kazuyoshi Z. Nanjo, Jun Izutsu, Yoshiaki Orihara, Toshiyasu Nagao, Seiya Uyeda

**Affiliations:** 1 Global Center for Asian and Regional Research, University of Shizuoka, 3-6-1, Takajo, Aoi-ku, Shizuoka 420-0839, Japan; 2 Institute of Oceanic Research and Development, Tokai University, 3-20-1, Orido, Shimizu-ku, Shizuoka 424-0902, Japan; 3 International Digital Earth Applied Science Research Center, Chubu Institute for Advanced Studies, Chubu University, 1200 Matsumoto-cho, Kasugai-shi, Aichi 487-8501, Japan; 4 Japan Academy, 7-3-2, Ueno Koen, Taito-ku, Tokyo 110-0007, Japan

**Keywords:** earthquake prediction, foreshocks, seismicity

## Abstract

The relation between the size of an earthquake mainshock preparation zone and the magnitude of the forthcoming mainshock is different between nucleation and domino-like cascade models. The former model indicates that magnitude is predictable before an earthquake’s mainshock because the preparation zone is related to the rupture area. In contrast, the latter indicates that magnitude is substantially unpredictable because it is practically impossible to predict the size of final rupture, which likely consists of a sequence of smaller earthquakes. As this proposal is still controversial, we discuss both models statistically, comparing their spatial occurrence rates between foreshocks and aftershocks. Using earthquake catalogs from three regions, California, Japan, and Taiwan, we showed that the spatial occurrence rates of foreshocks and aftershocks displayed a similar behavior, although this feature did not vary between these regions. An interpretation of this result, which was based on statistical analyses, indicates that the nucleation model is dominant.

## 1. Introduction

There is an intriguing discussion about the earthquake preparation process. Two major hypotheses proposed for this process are contradictory in the context of earthquake prediction. One of them is the most commonly cited cascade model [1,2]. In this model, the rupture initiates as a small slip on a small fault patch and continues to rupture further across a fault plane as long as the conditions are favorable. This model implies that small earthquakes begin in the same manner as large earthquakes. Because the rupture seems the sequence of small earthquakes, it is substantially impossible to predict the final rupture size, i.e., the magnitude of an earthquake. The second hypothesis is termed the nucleation model. The basic concept of this model is that the rupture process is to some degree deterministic and predictable [3], has implications for the whole rupture process, but manifesting the initial phase of the seismic wave at near-source stations. Olson and Allen [4] reported support the nucleation model by showing that the frequency content of radiated seismic energy within the first few seconds of rupture can be used to estimate the final magnitude of an earthquake.

Using southern California earthquake data, Lippiello et al. [5] statistically analyzed the linear density probability of the occurrence of earthquakes before and after small and intermediate mainshocks with magnitudes ranging between *m* = 2 and 5, where *m* is mainshock magnitude. They employed the mainshock identification method proposed by Felzer and Brodsky [6]. By extracting foreshocks and aftershocks within 12 hours before and after the mainshock, they calculated the linear density probability of the occurrence of foreshocks and aftershocks [5]. The most notable feature of their conclusion was that the shape of the linear density probability distribution was related to the magnitude of the mainshock. This implies that the rupture area deduced from the aftershocks and foreshock spatial distributions, which is possibly the earthquake preparation area, are almost identical.

Applying the approach based on Lippiello et al. [5] to Japanese seismicity, Nanjo et al. [7] also noted that the decay of linear density probability *ρ* with distance Δ*r* between the epicenters of the mainshock and foreshock/aftershock was well modeled by an inverse power law *ρ*(Δ*r*) ~ Δ*r*^−^*^η^* at Δ*r* >> 0, where *η* is a constant between 1 and 2 [8]. Nanjo et al. [7] also detected the largest point from the power law (scaling) relation. These authors defined this largest point (a peak the distribution) as the characteristic distance Δ*r*_c_, below which scaling was no longer valid due to large variance of seismicity density or low seismicity density at very short distances to the mainshock epicenter. They showed an increasing trend of Δ*r*_c_ with the mainshock magnitude classes *m* ∈ [*M*, *M* + 1), where *M* is magnitude, and Nanjo et al. used *M* = 3, 4, and 5, and compared it with the scaling relation between asperity size and *M*. Asperity size is based on the characteristic asperity radius, *l*_a_. The relation between *l*_a_ and *M* is obtained when the scaling between asperity area *S*_a_ and *M* are used [9], and by assuming that a circular asperity *S*_a_ = π*l*_a_^2^, they noted that the Δ*r*_c_-*m* correlation appears to be similar to the *l*_a_-*M* relation, interpreting Δ*r*_c_ as an indication of *l*_a_. 

In this study, we employ the method of Lippiello et al. [5] to analyze the linear density distributions *ρ*(Δ*r*) of foreshocks and aftershocks for three seismically active regions: Japan, California, and Taiwan. Focusing especially on the Japanese region, using inland and offshore earthquakes, we then investigated the spatial organization of earthquakes with small to intermediate size. This expands previous studies [5,7] that were limited by having only used inland earthquakes.

## 2. Method

In this study, we used earthquakes listed in the southern California earthquake catalog (SHLK catalog) [10] that was used by Lippiello et al. [5], the Japan earthquake catalog maintained by Japan Meteorological Agency (JMA), and the Taiwan earthquake catalog maintained by the Central Weather Bureau (CWB). The SHLK catalog is available in the form of an improved catalog of earthquake locations using waveform cross-correlation and cluster analysis. The dataset used for our analysis includes earthquakes from 1 January 1984 to 31 December 2002 in the range of 31 to 37° North and 114 to 121° West. For the JMA catalog, we initially considered earthquakes from 1 January 1995 to 3 May 2016 in the range of 122 to 146° East and 23 to 46° North with a depth shallower than 70 km. From this catalog, we then decided to use two datasets: one includes inland earthquakes having magnitudes *M* ≥ 2, and the other includes offshore earthquakes with *M* ≥ 3. The time period we considered for the CWB catalog was from 1 January 1991 to 29 February 2016. The study region was in the range of 117 to 128° East and 18 to 27° North with depths shallower than 70 km. From this study region, we selected and used earthquakes with *M* ≥ 3 in and around the Taiwanese mainland. For each of the catalogs, we conducted a completeness analysis and confirmed that we had used complete datasets. 

Following the method used by Felzer and Brodsky [5] and Lippiello et al. [6], we considered mainshocks as an event is identified as a mainshock if a larger earthquake does not occur in the previous *y* days and within a distance *L* (Table 1). In addition, a larger earthquake must not occur in the selected area in the following *y*_2_ days. The values of the parameters *L*, *y*, and *y*_2_ were selected as 100 km, 3 days, and 0.5 days, respectively, identical to those used elsewhere [5,6]. For mainshock magnitude class *m* ∈ [*M*, *M* + 1), we considered a linear density probability *ρ*(Δ*r*), which we defined as the number of aftershocks (foreshocks) in the succeeding (preceding) time interval (δ*t* = 12 h) with epicenters at a distance in the interval [Δ*r*, 1.2Δ*r*] from the mainshock, divided by 0.2Δ*r* and by their total number, i.e., the linear density [6,8] divided by the total number of identified aftershocks (foreshocks). Until this point, the theoretical development was the same as that employed by Lippiello et al. [5], except that those authors fixed Δ*r*_max_ = 3 km. We did not use Δ*r*_max_ = 3 km because the range of mainshock magnitudes considered in our study was wider than that considered by Lippiello et al. [5]. We used the formula, Δ*r*_max_ = 100 × *L_f_* (km), where *L_f_* is the fault length obtained by substituting *M* into the equation between *L_f_* and *M*: log *L_f_* = 0.6*M* − 2.9 [11]. By applying this method to the four datasets, and then for each dataset, Δ*r*_c_ values for different mainshock magnitudes were computed.

## 3. Results and Discussion

**Figure 1:**
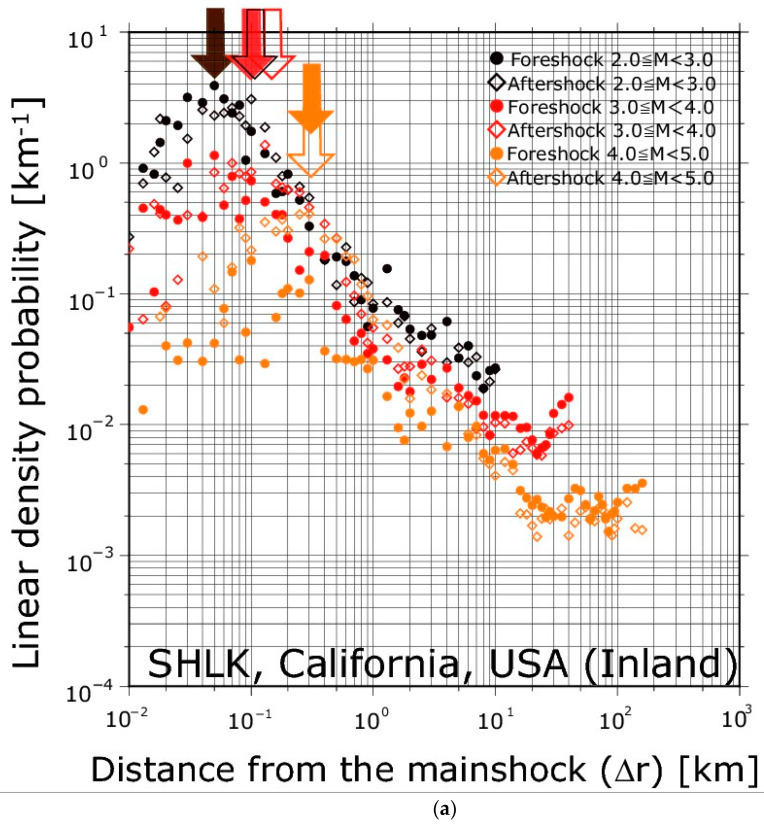

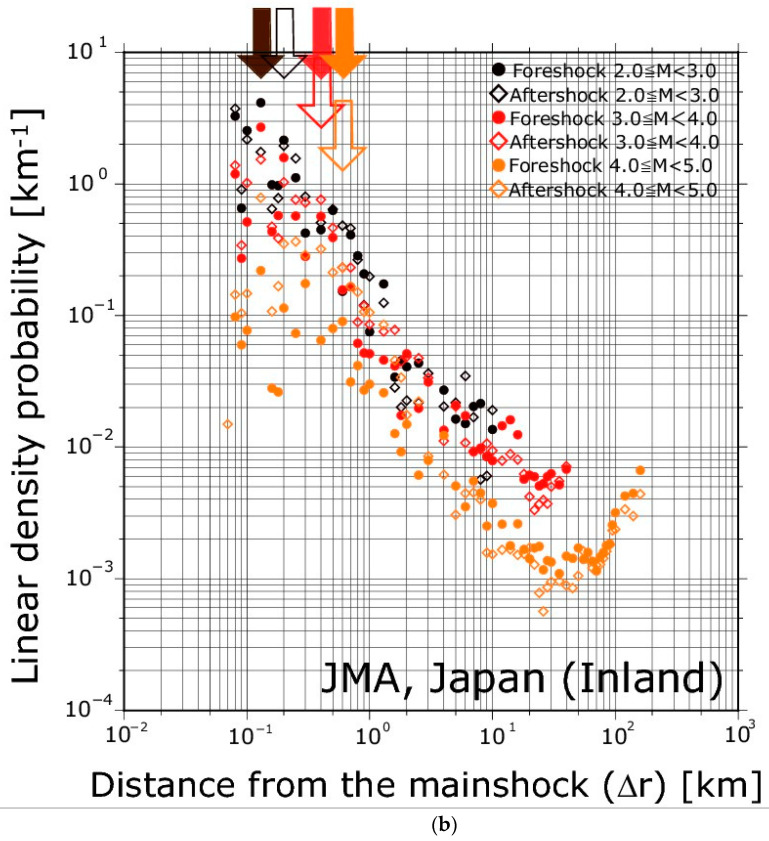

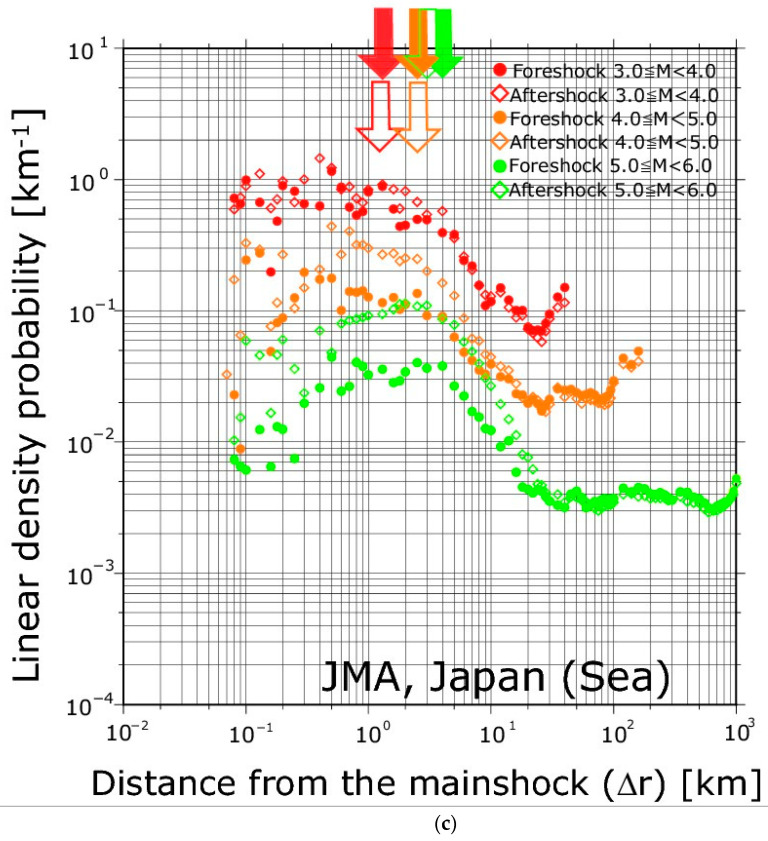

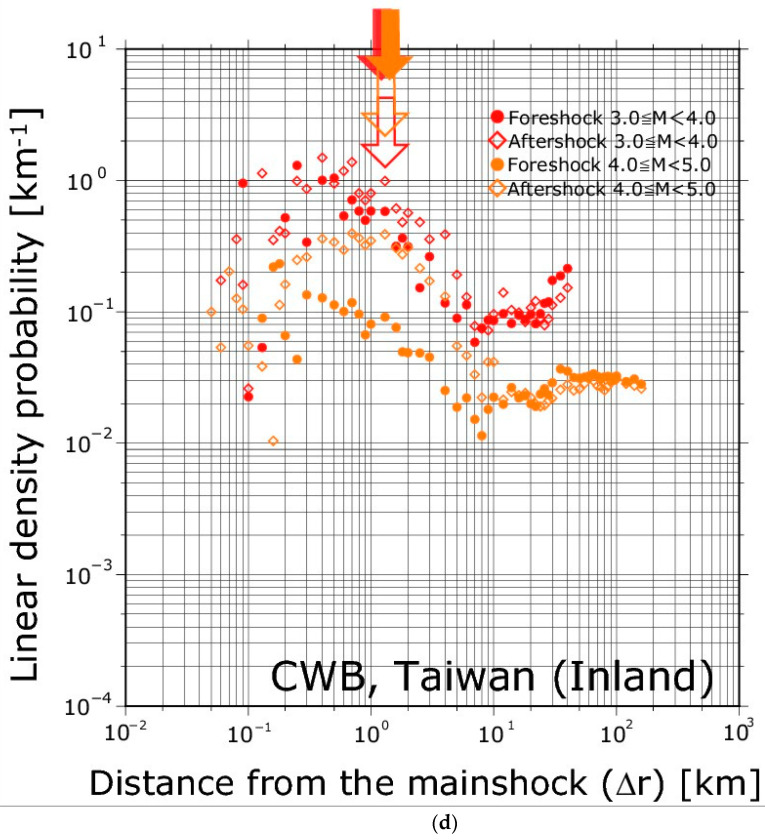
The linear density distributions ρ(Δ*r*) of foreshocks (circle) and aftershocks (diamond) for the four datasets: (**a**) California, (**b**) Japanese inland, (**c**) Japanese offshore, and (**d**) Taiwan. ρ(Δ*r*) is plotted for mainshocks in different ranges of mainshock magnitude class (different colors) *m* ∈ [*M*, *M* + 1), where *M* = 2, 3, 4 in a and b, *M* = 3, 4, 5 in c, and *M* = 3, 4 in d. Filled and blank arrows indicate Δ*r*_c_ eliminate determined by ρ(Δ*r*) of foreshocks and aftershocks, respectively.

Figure 1 shows the linear density probability of earthquakes occurring before and after small mainshocks obtained from Southern California (SHKL), Japan (JMA, inland and offshore), and Taiwan (CWB) earthquakes. All regions show a convex shape with a single peak. The result of the Southern California region (Figure 1a) closely coincided with the results obtained by Lippiello et al. [5]. Curiously, even the results for Japan (both inland and offshore) and Taiwan showed a similar tendency to the results obtained by Lippiello et al. [5]. 

The monomodal convex shape in Figure 1 consists of three parts. In the area, which was smaller than Δ*r*_c_, observed in other studies [5,7], each point of the distribution had a larger variance relative to the trend. On the other hand, in the region larger than Δ*r*_c_, *ρ*(Δ*r*) attenuated, following a clear power law. In addition, *ρ*(Δ*r*) displayed a nearly constant fluctuation when the value of Δ*r* was much larger. This feature, namely a nearly constant *ρ*(Δ*r*), can be regarded as a region beyond the earthquake preparation area. The area of smaller than Δ*r*_c_, possibly corresponding to slip areas, would be randomly generated seismicity, which yields increase or nearly constant of the linear density probability.

**Figure 2:**
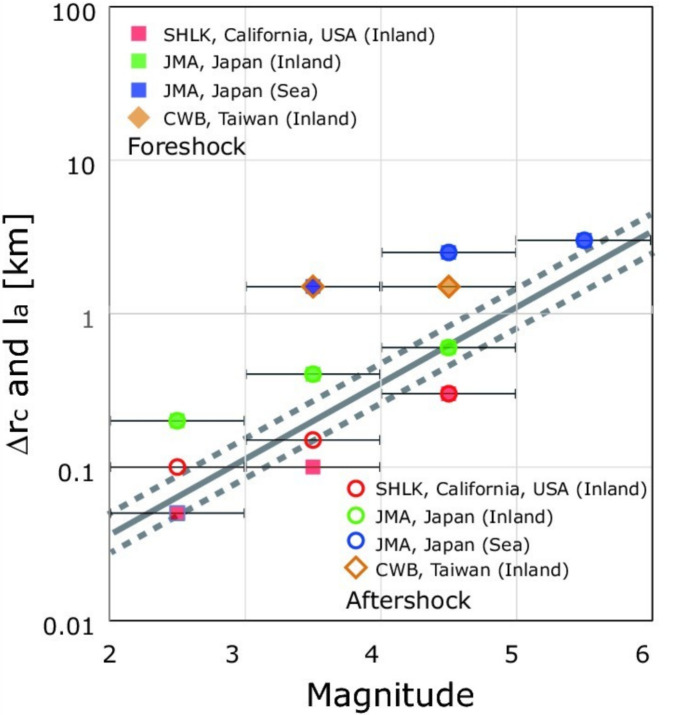
Plot of Δ*r*_c_ as a function of mainshock magnitude class *m* for foreshocks (filled symbols) and aftershocks (open symbols) for different datasets: California (red), Japan inland (green), Japan offshore (blue), and Taiwan (orange). The solid line was drawn by extrapolating from the scaling of the characteristic asperity radius *l_a_* with *M*, based on Skarlatoudis et al. [9]. One standard deviation limits are shown by a dashed line.

Figure 2 shows the relationship between Δ*r*_c_ and mainshock magnitude *m*. If we assume a circular asperity (*S*_a_ = π*l*_a_^2^), where *l*_a_ is a characteristic asperity radius [9], the *l_a_*-*M* line correlates well with the Δ*r*_c_-*m* correlation to be obtained from this study. 

Skarlatoudis et al. (2005) used moment magnitude, and not local magnitudes used for Japan, California, and Taiwan, to obtain the scaling shown in Figure 2. We checked the conversion to the moment magnitude from the local magnitudes for California [12], Japan [13,14,15], and Taiwan [16,17], and confirmed that the similarity between the Δ*r*_c_-*m* correlation and the *l*_a_-*M* relation is valid, supporting our result. 

The properties of the earthquake preparation process in California, Japan, and Taiwan (Figure 1 and Figure 2) are similar to those observed in previous studies [5,7,18,19]. Given the widely differing tectonic conditions that cause different types of faults, one might expect strong differences in the preparation process for major ruptures. However, quantitatively documented spatial organization was found in all of these areas (Figure 1). Moreover, they have a common feature characterized by the Δ*r*_c_-*m* correlation, which is similar to the *l*_a_-*M* relation (Figure 2). This observation suggests that this type of foreshock organization exists, as was also pointed out by Lippiello et al. [19], and should be investigated more fully. 

We point out that our finding is consistent with the Critical Point (CP) earthquake hypothesis ([20,21,22,23]), where failure in the crust is a scaling up process in which stress spreads out over the entire area before the mainshock. The next step would be to apply our method to investigate the accelerating moment release and the growth of spatial correlation length with respect to their predictive power.

According to Schorlemmer et al. [24], there was the clear relation between the stress and the seismicity expressed by *b*-value, which indicated that the linear density probability might depend on the stress. At the boundary Δ*r*_c_, the stress distribution, therefore, was different. In addition, in the context of self-organized critical process possibly related to the domino-like model, Yoshioka and Sakaguchi [25] showed that widely uniform stress was distributed before the large avalanche in the photoelasticity sandpile experiments, which is rather similar to the nucleation model. These [24,25] agreed with our results. In other words, the uniform stress inside the Δ*r*_c_, i.e., a part of final rupture area, was accumulated before the mainshock. Although the study of initial seismic waveform for understanding earthquake preparation process [1,2] might not provide the information of the final rupture area, our present results showed that the foreshock seismicity provided the information of the final rupture area.

## 4. Conclusions

The spatial distributions of seismicity density for foreshocks and aftershocks, characterized by *ρ*(Δ*r*), are similar to each other, and this behavior is observable regardless of different earthquake catalogues (Figure 1). We focused on earthquakes immediately before and after a mainshock relatively near the mainshock hypocenter (see the “Method” section), so the spatial distributions of foreshocks and aftershocks were associated with the earthquake preparation area and the rupture area, respectively. Furthermore, we found that the spatial distributions of foreshocks and aftershocks were associated with the characteristic asperities radius, *l*_a_. This indicates that the earthquake preparation and rupture areas grew as the magnitude of the mainshock increased, the latter being scaled with *l*_a_ [9]. Our observation shows that the size of the area fractured during the mainshock as well as the magnitude of the mainshock are encoded in the foreshock spatial organization [5,18,19]. 

As described in the “Introduction” section, two major hypotheses of the preparation process have been proposed: the nucleation model [3,4] and domino-like cascade models [1,2]. Conventionally, both models pertain to individual fault ruptures, but not to ruptures that preceded other (possibly larger) ruptures. We assumed an alternative approach by utilizing foreshocks (small ruptures) that are considered to be the manifestation to the preparation process that preceded mainshocks (eventual ruptures). We then showed their association with the size of the mainshock and its magnitude (Figure 2). In other words, the magnitude of the mainshock is to some degree deterministic before completing eventual rupture propagation. Thus, our preferred model is the nucleation model rather than the domino-like cascade model.

## Figures and Tables

**Table 1:** Number of selected mainshocks.

Magnitude Ranges of Mainshocks	SHLK, California, USA (Inland)	JMA, Japan (Inland)	JMA, Japan (Sea)	CWB, Taiwan (Inland)
2 ≤ *m* < 3	6275	10,601	-	-
3 ≤ *m* < 4	1704	3512	25,632	2098
4 ≤ *m* < 5	205	719	9609	1254
5 ≤ *m* < 6	-	-	1780	-
